# Impact of a remote nutrition education on low-carbohydrate diet based in type 2 diabetes management: findings from a Brazilian primary care randomized controlled trial

**DOI:** 10.1007/s13340-026-00898-2

**Published:** 2026-04-22

**Authors:** Gisely Sanagiotto Balbinot, Romulo Scariot Costódio, Geraldo Emílio Vicentini

**Affiliations:** 1 Master’s Program in Applied Health Sciences, Health Sciences Center, State University of Western Paraná (UNIOESTE), Rodovia Vitório Traiano, Km 2, no 730, Bloco I, Sala 02, Bairro Água Branca, Francisco Beltrão, PR CEP 85.601-970 Brazil; 2 Health Sciences Center, State University of Western Paraná (UNIOESTE), Rodovia Vitório Traiano, Km 2, no 730, Bloco I, Sala 02, Bairro Água Branca, Francisco Beltrão, PR CEP 85.601-970 Brazil

**Keywords:** Type 2 diabetes mellitus, Diet, Smartphone, Education, Primary health care

## Abstract

**Introduction:**

The difficulty of nutritional management of type 2 diabetes mellitus (T2DM) in primary health care in Brazil highlight the need for innovative strategies based on nutritional education.

**Methods:**

This study evaluated the effectiveness of an online educational intervention based on a low-carbohydrate diet for adults with T2DM and compared with conventional primary care management. A 16-week parallel randomized clinical trial enrolled 58 non-insulin users (mean age: 61 years), randomized to an Intervention (*n* = 29) or Control group (CG) (*n* = 29).

**Results:**

After 16 weeks, the Intervention Group (IG) showed significant reductions in HbA1c (− 0.91 ± 0.16%), fasting glucose (− 1.46 ± 0.06 mmol/L) and BMI (− 1.63 ± 0.8 kg/m²). In contrast, the CG exhibited significant increases in fasting glucose (+ 1.03 ± 0.17 mmol/L) and Body Mass index (+ 0.96 ± 0.5 kg/m^2^), with no significant HbA1c change (+ 0.25 ± 0.04%). Between-group comparisons favored the IG (*p* < 0.001), with net differences of 1.17% in HbA1c and 27.5% in fasting glucose. Additionally, 31% of IG participants achieved HbA1c ≤ 6.5%, whereas none of the CG participants achieved the HbA1c target. Use of oral antidiabetic medication decreased significantly in the *IG*; notably, this reduction was not observed in the *CG* (44.8% vs. 0%, *p* < 0.001).

**Conclusion:**

In conclusion, the online education intervention improved glycemic control, reduced BMI, and lowered medication requirements, representing a promising strategy for T2DM management with potential to reduce complications and healthcare system costs.

## Introduction

Type 2 diabetes mellitus (T2DM) accounts for the majority of diabetes cases and poses a major global public health challenge due to its increasing prevalence and substantial economic burden on healthcare systems worldwide [1, 2]. The International Diabetes Federation has projected that the number of individuals with diabetes will reach 783 million by 2045 [3]. Recent data from Brazil indicate that the adult population in the country’s capital cities has a T2DM prevalence close to 10% [1]. Approximately 50% of Brazilians are covered by Primary Health Care (PHC) services [4], and the Unified Health System (SUS) provides around 30 million diabetes-related appointments each year, highlighting the clinical and economic burden of the disease nationwide [1, 5].

The T2DM is a progressive metabolic disorder characterized by chronic hyperglycemia resulting from the failure of pancreatic β-cells to compensate for peripheral insulin resistance, a process frequently driven by ectopic lipid accumulation and a complex interplay between genetic predisposition and environmental factors [6]. Chronic complications associated with T2DM — such as nephropathy, neuropathy, ischemic heart disease, and cerebrovascular disease — significantly reduce life expectancy; however, proper treatment and glycemic control effectively lower morbidity and mortality related to T2DM [7, 8].

The prevalence of diabetes-related complications in Brazil disproportionately affects people with lower income and educational levels, underscoring the need for better-qualified PHC services to serve this population [4]. In PHC, chronic conditions such as T2DM are typically managed through short medical consultations focused on drug prescription [9], revealing gaps in care delivery within Basic Health Units and frequently resulting in unsatisfactory diabetes outcomes [10].

The role of health professionals is crucial in the management of diabetes, particularly regarding dietary behavior. Changing eating habits is challenging for patients and requires consistent professional support to enable essential behavioral changes. Nutritional education is therefore a key intervention for diabetes management, as inadequate dietary patterns are a major cause of poor glycemic control [11].

Low-carbohydrate dietary interventions, even when delivered via remote care platforms have shown excellent results in metabolic disorders associated with insulin resistance, particularly T2DM, where they have been effective in improving glycemic control through significant reductions in HbA1c and antidiabetic medication use [12, 13].

Health professionals play an essential role in encouraging, monitoring, and supporting behavioral change; frequent communication and structured educational interactions have been shown to be effective [14]. Remote strategies, including mobile applications and digital tools, have also demonstrated benefits for nutritional education, glycemic monitoring, and behavioral support [12, 15].

Despite growing evidence supporting dietary strategies, the effectiveness of structured online nutritional education as a scalable approach within primary health care remains underexplored. The aim of this study was to investigate, within the context of primary health care, the effectiveness of an online nutrition education intervention focused on a low-carbohydrate diet for individuals with T2DM, compared with standard primary care management after 16 weeks. The intervention was delivered using structured digital educational materials on nutrition education and meal planning, combined with continuous professional support, to encourage the adoption of healthier habits and promote glycemic control. To our knowledge, this study represents the pioneering clinical trial to evaluate a remote nutritional education intervention utilizing a low-carbohydrate approach within the Brazilian primary care setting.

## Materials and methods

### Study design and participants

**Fig. 1:**
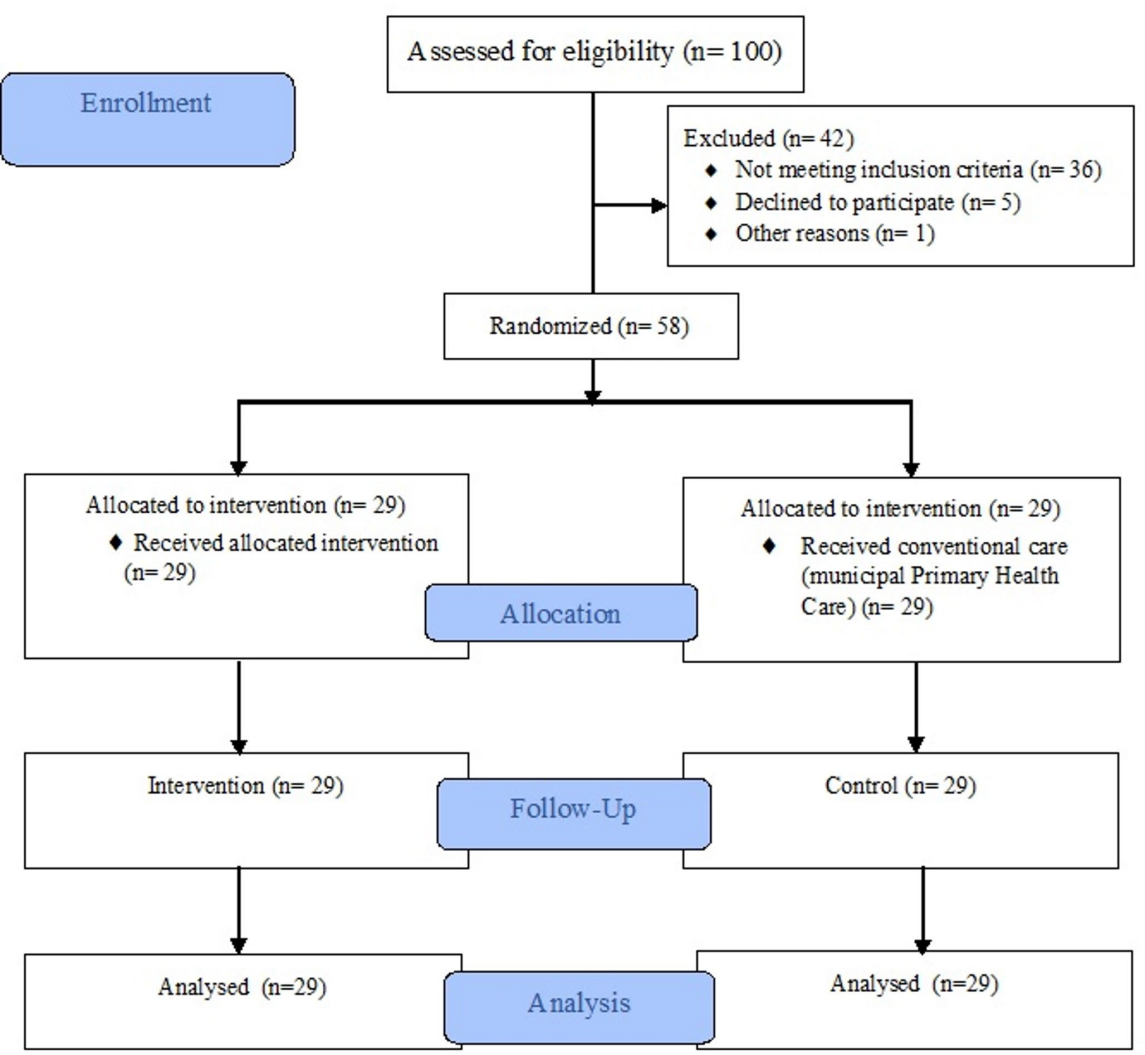
CONSORT Flowchart of the selection of study participants. n = 100 refers to the initial list of patients diagnosed with type 2 diabetes mellitus, provided by the municipal health service

Patients diagnosed with T2DM were recruited from a centralized municipal registry of individuals receiving care within the Primary Health Care network of Francisco Beltrão, Paraná, Brazil. The registry comprised a list of 100 eligible patients provided by the primary care team. Recruitment was carried out through phone calls and a smartphone messaging application. A total of 58 participants were included in a 16-week nutrition education program according to the following inclusion criteria: adults aged 40–89 years; diagnosis of non-insulin-treated T2DM; baseline HbA1c ≥ 6.5%; ability to read and understand Portuguese (Brazil); full access to the internet; and use of a smartphone communication app (individually or in groups) (Fig. 1).

Exclusion criteria were as follows: diagnosis of other types of diabetes; previous bariatric surgery; severe cardiovascular disease; chronic kidney disease limiting participation; pregnancy or lactation; eating disorders; or enrollment in weight-loss programs.

This was a single-center, prospective, 16-week randomized controlled trial conducted between August and November 2024. Participants (*n* = 58) were randomly assigned, following simple randomization procedures (random numbers generated using Excel software, version 2511), to one of the two arms of this study: Intervention Group (IG) or the Control Group (CG). Allocation was performed by the principal investigator prior to accessing the electronic health records. Clinical data were obtained only after group assignment, ensuring allocation concealment and minimizing selection bias. Clinical and laboratory data were obtained from WINSaúde, the official electronic record platform of the municipal health system. Data were collected at baseline and after 16 weeks of intervention.

## Sample size calculation

The sample size was calculated using the formula for superiority trials described by Zong [16], with change in HbA1c defined as the primary outcome, and informed by previously published studies reporting significant reductions with comparable interventions [17, 18]. An expected mean reduction of 0.2 ± 0.5% in the CG and 0.6 ± 0.5% in the IG was assumed. Using α = 0.05 and a power of 0.80 to detect clinically relevant between-group differences, a sample size of 25 participants per group was determined. Considering an expected dropout rate of 15%, 30 participants per group were enrolled.

## Ethics

The study was approved by the Research Ethics Committee of the Universidade Estadual do Oeste do Paraná (UNIOESTE) (Date of approval: 05/26/2024; approval no. 6.849.418; CAAE 80074824.2.0000.0107). All participants were informed about the study objectives and procedures and provided written informed consent before enrollment. This study adhered to the Consolidated Standards of Reporting Trials (CONSORT) guidelines and was registered at the Brazilian Clinical Trials Registry (ReBEC) under identifier RBR-39xwyzr.

## Intervention

### Intervention group

In this group, nutritional education strategies were implemented based on a low-carbohydrate dietary approach. The educational sessions were delivered entirely remotely through a smartphone messaging application (WhatsApp). Participants also received a visual nutrition guide provided in digital format (PDF).

Once participants were provided with nutritional literacy regarding the nature and sources of carbohydrates, participants followed a dietary strategy that limited the intake of carbohydrate-rich foods to approximately four tablespoons per meal. This approach focuses on reducing total carbohydrate volume through the selection of foods with low carbohydrate density, rather than strict daily gram quantification. By utilizing familiar household measures, we aimed to facilitate participant comprehension and promote dietary autonomy in food choices [19]. This pragmatic approach was intentionally selected to reflect real-world conditions within the primary health care system. The nutritional education program encouraged the consumption of unprocessed meats, non-starchy vegetables, whole dairy products, nuts, and seeds, and, depending on individual tolerance, small portions of fruits and legumes [20].

The intervention lasted 16 weeks and was delivered entirely remotely. During the intervention period, participants received nine educational videos lasting between 15 and 50 min, developed and presented by the study’s nutritionist and principal investigator. The videos were recorded as expository lectures in which the researcher appeared on screen alongside slides containing key concepts and illustrative images, using a split-screen format. Recordings were made using a smartphone and ring light to ensure adequate sound and image quality. The videos were publicly available on a professional YouTube^®^ channel containing only educational material, without any personal or identifiable data from participants, ensuring unrestricted access and reproducibility of the educational content.

The video lessons were developed in accessible language and with a supportive tone, progressively addressing: (1) an introduction to T2DM, its pathophysiology, and complications; (2) the importance of nutrition in metabolic control, focusing on macronutrients, micronutrients, and energy metabolism; (3) practical guidance for implementing a low-carbohydrate diet in daily life; and (4) label reading and interpretation of food packaging, emphasizing misleading sugar descriptions that may confuse people with diabetes.

Participants in the IG also received a digital visual nutrition guide designed by the nutritionist, accompanied by an additional explanatory video detailing its content and practical application, reinforcing the main concepts of nutrition education within the low-carbohydrate framework of this intervention.

To maintain adherence throughout the 16-week program, participants received three educational messages per week via WhatsApp, totaling 48 messages, including: (1) specific dietary recommendations for the IG, with practical examples of meals, food substitutions, and balanced macronutrient combinations; (2) motivational messages, one to two per week, encouraging dietary adherence, glycemic control, and self-care; (3) continuous support through individual feedback and clarification of doubts directly with the study’s nutritionist, who remained available for ongoing guidance and to address potential side effects related to the low-carbohydrate diet.

Participants in this group were encouraged to maintain their usual routines, including physical and recreational activities. They were instructed to report any significant deviations from their habitual activity levels to the research team. Aside from monitoring these potential shifts, no specific interventions or structured physical activity assessments were implemented, thereby maintaining the group’s ‘real-world’ baseline conditions.

### Control group

Participants in the CG did not receive a structured nutrition education program during the study period and had contact with the research team during recruitment. They were also invited by the research team and contacted by phone to schedule and carry out the procedures of this research within the stipulated timeframe. They continued to receive the conventional care provided by municipal Primary Health Care (PHC) units, which include by general guidelines for T2DM management, prevention, early diagnosis, and continuous monitoring through a multidisciplinary team offering personalized and preventive care. This standard approach includes general guidance on healthy eating, physical activity, regular blood glucose monitoring, and proper medication use, in accordance with the Sociedade Brasileira de Diabetes guidelines [2]. However, this routine management does not involve structured follow-up meetings or remote support. Healthcare procedures vary significantly across individual primary care units. Typically, patients are evaluated every three or six months, although this frequency may be adjusted based on specific unit protocols. Consultations and follow-ups are conducted by physicians and nurses; consequently, nutritional advice is delivered by these professionals, as clinical nutritionists are not permanently stationed at every primary health unit within the municipality. This study reflects the specific public health infrastructure of our municipality, acknowledging that these conditions may not represent the national average but provide critical localized data. We deliberately chose not to monitor the procedures in PHC. This was a strategic methodological decision to ensure that the CG represented real-world clinical practice without interference. By maintaining this distance, we avoided the Hawthorne effect—where the quality of service or patient behavior might improve simply due to the awareness of being evaluated.

## Assessments

Personal data, including age and sex, were collected, as well as laboratory data (fasting glucose and glycated hemoglobin [HbA1c]) and anthropometric parameters (body weight, height, and body mass index [BMI], calculated as weight divided by height squared).

Anthropometric and laboratory data—including fasting glucose and HbA1c—were extracted from electronic medical records for both groups at baseline and week 16. To ensure comparability and reliability, all measurements were obtained within a maximum two-week window from the scheduled evaluation points. Participants were invited by the research team to complete these procedures at the Primary Health Care (PHC) units during this specific timeframe.

The primary outcome was the variation in glycemic control, assessed by the change in HbA1c levels after 16 weeks. Secondary outcomes included the proportion of participants who achieved HbA1c ≤ 6.5%, as well as changes in anthropometric parameters and antidiabetic medication use. The study aimed to assess real-world applicability within the local primary care context, identifying practical and sustainable changes in patients’ dietary and metabolic profiles.

Decisions regarding the initiation, adjustment, or discontinuation of antidiabetic medications were made exclusively by the patients’ treating physicians within the public health care system, according to routine clinical practice rather than a study-specific protocol. The research team did not interfere with therapeutic decisions at any time. Information on medication use was extracted from electronic health records at baseline and after the 16-week intervention.

### Statistical analysis

Data were analyzed using IBM SPSS Statistics for Windows, version 21.0 (IBM Corp., Armonk, NY, USA). Continuous variables were expressed as mean ± standard deviation (SD). Normality was verified using the Shapiro–Wilk test. Categorical variables were expressed as absolute (n) and relative (%) frequencies. Within-group comparisons (baseline vs. post-intervention) were performed using Student’s paired *t*-test or the nonparametric Wilcoxon signed-rank test. Between-group comparisons (Δ = post – pre) were analyzed using Student’s independent *t*-test or the nonparametric Mann–Whitney *U* test. Changes in antidiabetic medication use were evaluated using Pearson’s chi-square or Fisher’s exact test. A significance level of 5% (*p* < 0.05) was adopted.

## Results

### Participant characteristics

Of the individuals included in the study, 58 were randomly assigned to the intervention and control groups. The baseline characteristics of the 58 participants in the IG and CG are presented in Table 1. No statistically significant differences were observed between groups regarding age, sex, height, body weight, body mass index (BMI), glycated hemoglobin (HbA1c), or fasting glucose levels, indicating homogeneity at the beginning of the study.

**Table 1:** Baseline characteristics of participants

Characteristic	CG (*N* = 29)	IG (*N* = 29)	*p*-value
^#^Age (years)	62.9 ± 11.1	60.4 ± 11.5	0.4013
^♦^Sex M/F % (N)	58.6% (17) / 41.4% (12)	51.7% (15) / 48.3% (14)	0.7918
^♣^HbA1c (%)	8.6 ± 1.6	7.9 ± 1.0	0.0511
^♣^Fasting Glucose (mmol/L)	8.58 ± 2.80	9.37 ± 2.41	0.1024
^#^Weight (kg)	76.8 ± 15.6	85.2 ± 17.5	0.0599
^#^BMI (kg/m^2^)	29.2 ± 7.6	31.4 ± 7.5	0.2837

^∗^ Statistically significant

^#^ Student’s t test

^♣^Test U of Mann-Whitney

^♦^Test Χ^2^

### Primary outcome

Of the 58 participants who completed the study, 29 were assigned to each group at the end of the 16-week period. The primary outcome was defined as the change in HbA1c levels from baseline to week 16, and fasting glucose, body weight, body mass index (BMI), and use of antidiabetic medication as secondary outcomes. Baseline, final, and variation (Δ) mean values are shown in Table 2.

After 16 weeks, the *IG* demonstrated improved glycemic control, with significant reductions in HbA1c (mean decrease of 0.91%) and fasting glucose (mean decrease of 15.5%). In contrast, the *CG* exhibited a significant increase of 12% in fasting glucose (*p* = 0.019), while the slight rise in HbA1c was not statistically significant. Between-group comparisons after the intervention period revealed a significantly greater improvement in glycemic control among participants in the IG compared with those in the CG, with net percentage differences of 1.17% for HbA1c and 27.5% for fasting glucose at the end of the experimental period (Table 2).

**Table 2:** Effectiveness of Nutritional education on clinical outcomes in the intervention and control groups and the within-group variation after 16 weeks

Variable	Control group (*n* = 29) (mean ± SD)			Intervention group (*n* = 29) (mean ± SD)			
	Baseline	Final	Ξ*p*-value	Baseline	Final	Ξ*p*-value	✝*p*-value
HbA1c (%)	8.62 ± 1.65	8.87 ± 1.69	0.3038	7.90 ± 1.04	6.99 ± 0.88	< 0.001^∗^	< 0.001^∗^
Change	+ 0.25 ± 0.04			− 0.91 ± 0.16			
Fasting glucose (mmol/L)	8.58 ± 2.80	9.62 ± 2.97	0.019*	9.37 ± 2.39	7.92 ± 2.33	0.0004^∗^	< 0.001^∗^
Change	+ 1.04 ± 0.17 (+ 12.1%)			− 1.46 ± 0.06 (-15.5%)			
Body weight (kg)	76.8 ± 15.6	79.4 ± 16.9	0.0002*	85.2 ± 17.5	80.9 ± 15.9	< 0.001^∗^	0.001^∗^
Change	+ 2.53 ± 1.3 (+ 3.3%)			− 4.35 ± 1.6 (5.1%)			
BMI (kg/m^2^)	29.2 ± 7.6	30.2 ± 8.1	0.0002*	31.4 ± 7.5	29.7 ± 6.7	< 0.001^∗^	0.001^∗^
Change	+ 0.96 ± 0.5 (+ 3.3%)			− 1.63 ± 0.8 (-5.2%)			

✝For between-group comparisons; Ξfor within-group comparisons

^*^Statistically significant

SD = Standard Deviation; HbA1c = Glycated Hemoglobin; BMI = Body Mass Index

### Secondary outcomes

The results for the secondary outcomes are presented in Table 2. Significant reductions were observed in anthropometric parameters, with decreases of 5.1% in body weight and 5.2% in BMI in the IG. Conversely, the CG showed significant increases of 3.3% in both body weight and BMI. Between-group comparisons revealed significant net differences of 8.4% in body weight and 8.5% in BMI, highlighting the positive effect of the nutritional intervention on the evaluated metabolic outcomes and confirming that the intervention also yielded superior results for secondary outcomes in the management of T2DM in primary health care.

### Magnitude of the intervention effect

The magnitude of the differences observed within each group at the end of the intervention period, compared with baseline values, demonstrated the important clinical impact of the intervention in this study (Table 3). In this regard, when assessing the achievement of a glycemic target compatible with remission based on the reference value for glycated hemoglobin (HbA1c ≤ 6.5%), we observed that the *IG* was the only group in which participants reached this level of glycemic control (31% of patients), whereas no such cases were observed in the CG.

**Table 3:** Effectiveness of nutrition education based on low-carbohydrate diet from baseline and proportions of patients achieving HbA1c < 6.5% (glycemic target) in intervention and control groups after 16 weeks

Efficacy on HbA1c	Groups		
	IG (*n* = 29)	CG (*n* = 29)	*p*-value
% (n) of Participants who reduced HbA1c from baseline	93.1% (27)	31.0% (9)	< 0.001***
% (n) of Participants who achieved HbA1c < 6.5%	31.0% (9)	0% (0)	0.002*

***Statistically significant according to Fisher’s Exact Test

IG , Intervention group; CG, Control group; HbA1c,  Glycated hemoglobin

The IG also showed a high proportion (> 90%) of participants who achieved a significant reduction in HbA1c compared with baseline values, whereas the *CG*, which underwent conventional management, demonstrated a more limited response, with approximately 30% of participants showing improvement.

### Use of oral antidiabetic medications

Table 4 presents the changes in the use of oral antidiabetic medications throughout the study. A significantly higher proportion of participants in the IG showed reductions or discontinuation of medication use, and no participant required the addition or dose increase of any drug during the study period. In contrast, participants in the CG experienced additional or dose increases of antidiabetic medications, with no cases of reduction or discontinuation observed during the same period.

**Table 4:** Changes in oral antidiabetic medication over 16 Weeks of the experimental period

Change in medication	IG (*n* = 29) n (%)	CG (*n* = 29) n (%)	*p*-value
Medication reduced	6 (20.7%)	0 (0.0%)	< 0.001*
Medication discontinued	7 (24.1%)	0 (0.0%)	
Total reduced/discontinued	13 (44.8%)	0 (0.0%)	
Medication added	0 (0.0%)	9 (31.0%)	< 0.001*
Dose increased	0 (0.0%)	1 (3.4%)	
Total added/increased	0 (0.0%)	10 (34.4%)	
No changes	16 (55.2%)	19 (65.5%)	0.435

*Fisher’s exact test

## Discussion

The greater reduction in HbA1c, as well as the higher proportion of individuals achieving target HbA1c levels in the IG compared with the CG, highlights the effectiveness of this structured online nutritional education strategy. Since HbA1c is widely recognized as a key marker of long-term glycemic control, its reduction is known to correlate with a lower incidence and progression of microvascular and macrovascular complications, as demonstrated by studies emphasizing the benefits of early glycemic control [21].

T2DM remission has been extensively discussed in recent years, with several proposed definitions. Some authors define remission as maintaining HbA1c < 6.5% for ≥ 3 months without medication use [22]. This state is often accompanied by weight loss, as observed in the DIADEM-I trial [23]. Other authors propose broader criteria for T2DM remission, including reductions in medication burden, improvement of metabolic markers, and shorter periods required to achieve target HbA1c levels [24, 25].

The improvement in HbA1c levels observed in this study is consistent with previous research confirming the efficacy of structured nutritional interventions, particularly those that include nutrition education and carbohydrate restriction, in managing T2DM [26]. Tay et al. [27] reported significant HbA1c reductions when comparing this approach to low-fat diets, emphasizing that professional supervision was crucial for adherence. Similarly, Athinarayanan et al. [2] observed sustained improvements in remote low-carbohydrate interventions, where ongoing educational support played a key role in maintaining outcomes. More recently, researchers from La Trobe University [19] reported T2DM remission in a substantial number of participants, even without caloric restriction, reinforcing that macronutrient composition combined with professional guidance can be decisive for clinical success.

The HbA1c reduction observed in our study approached 1% in the IG, exceeding the magnitude reported in previous studies such as Tay et al. [27] who found a 0.6% decrease under similar conditions. The CG in our study showed a net increase in HbA1c exceeding 1%, comparable in magnitude to results from intensive in-person interventions such as the DiRECT trial [25]. These findings suggest that online nutrition education combined with low-carbohydrate dietary guidance can achieve clinically meaningful effects on glycemic control among patients managed in primary care, who often struggle with adherence to conventional diabetes treatment [28]. A meta-analysis showed that a mean 0.9% reduction in HbA1c was associated with a 9% relative reduction in major cardiovascular events (HR 0.91; 95% CI 0.84–0.99) and a 15% reduction in myocardial infarction relative risk (HR 0.85; 95% CI 0.76–0.94) [29], supporting the clinical significance of the results obtained in this study.

A recent meta-analysis reported that short-term interventions (≤ 6 months) consistently demonstrated rapid improvements in glycemic markers, particularly HbA1c and blood glucose levels, supporting low-carbohydrate diets as effective initial strategies for glycemic control in patients with T2DM. However, the sustainability of metabolic benefits beyond this period is variable and strongly dependent on long-term dietary adherence [30].

In the context of diabetes mellitus, improvements in glycemic control—as reflected by reductions in glycated hemoglobin—are consistently associated with clinically meaningful benefits. Even modest decreases of up to 0.5% in HbA1c are linked to reductions in cardiovascular events and mortality, whereas reductions approaching 1% correspond to an approximate 20% decrease in mortality risk and a 37% reduction in microvascular complications [15].

Fasting glucose is a key biomarker reflecting various aspects of carbohydrate metabolism, essential for both diabetes diagnosis and monitoring [31]. Markers of insulin secretion and sensitivity are particularly relevant for assessing the effectiveness of dietary interventions on glycemic control [32]. Previous studies, such as Saslow et al. [33]., demonstrated that low-carbohydrate interventions lead to significant improvements in fasting glucose — findings consistent with the results of our study. The combination of specific dietary guidance with online support appears to have enhanced adherence and consequently improved glycemic outcomes, demonstrating the effectiveness of the remote nutrition education model implemented here [34].

Beyond glycemic control, the intervention also positively affected secondary outcomes, improving anthropometric parameters such as body weight and BMI. Growing evidence indicates that significant weight loss contributes to T2DM remission and improved pancreatic β-cell function [23]. The weight loss observed in this study is clinically relevant within the T2DM context. International studies, such as the LOOK AHEAD trial, demonstrated that a 10% reduction in body weight was associated with a 21% reduction in cardiovascular disease risk [35]. The DiRECT study by Lean et al. [25] showed that intensive weight-loss interventions could induce T2DM remission in a substantial proportion of patients. The present study, using a remote education approach, achieved approximately 5.5% weight loss within 16 weeks — comparable to results from in-person interventions — while offering advantages of greater accessibility, continuity of care, and lower operational cost [36]. By adopting a low-carbohydrate dietary strategy, we encouraged participants to maintain adequate protein intake relative to caloric content, favoring higher protein consumption alongside a slight energy deficit. This combination has been shown to effectively support weight management by enhancing satiety, increasing energy expenditure and improving insulin sensitivity [37].

The CG in this study exhibited a slight, albeit unexpected, increase in body weight, while HbA1c levels remained unchanged. While this observation may have been influenced by baseline imbalances inherent to the randomization process—as previously addressed in the limitations—it is consistent with broader evidence regarding the challenges of diabetes management in primary care. National studies often highlight suboptimal outcomes in conventional care settings, where traditional strategies frequently fail to promote significant metabolic improvements [38]. Evidence suggests that while SUS users often present with poorer baseline glycemic control due to delayed access to specialized care—with waiting times reaching eight months—they exhibit remarkable clinical responsiveness once intervention is secured. This is evidenced by the more pronounced reduction in HbA1c levels observed among public health patients compared to those in private settings [39, 40]. It is important to emphasize that the objective of this research was not to audit public health services, but rather to propose evidence-based enhancements to the current care model. Our findings suggest that the conventional approach may be insufficient to counteract the progressive nature of T2DM, whereas the superior results observed in the IG underscore the potential of specialized, remote nutritional support to fill these persistent gaps in primary care.

Regarding oral antidiabetic medications, the intervention was associated with a higher proportion of participants reducing or discontinuing medication use compared with the CG. This finding suggests that improved glycemic control contributed to reduced pharmacological dependence, offering additional benefits such as fewer side effects and lower treatment costs — outcomes consistent with current discussions on T2DM potential remission strategies [12, 25].

Hallberg et al. [41]. observed that remote nutrition-based interventions could lead to substantial reductions in antidiabetic medication use, with 60% of participants reducing or stopping at least one drug. The results of the present study align with that evidence indicating decreased medication dependency and broader health benefits for patients.

The online format of the intervention demonstrated strong potential to expand access and optimize resources in primary care. Digital technologies have proven effective in delivering high-quality information, supporting self-care, and enhancing patient–professional communication [42] thereby complementing conventional care in a cost-effective way. Digital education interventions have shown substantial efficacy in improving glycemic control, body composition, treatment adherence, and patient satisfaction [43]. A systematic review confirmed that telemedicine interventions integrated with structured programs significantly improved HbA1c levels, emphasizing that successful implementation requires adequate local infrastructure and consistent patient–professional interaction [21].

The online format of the intervention may have played a central role in the outcomes observed. By increasing accessibility, enabling continuous professional support, and facilitating ongoing patient engagement, this model likely encouraged the adoption of healthier habits while reducing common barriers associated with usual care. The intervention offered nutritional guidance and appropriate food choices aligned with the participants’ clinical needs. Effective glycemic control and clinically significant weight loss are primarily driven by patient-centered educational strategies that bridge the gap between clinical guidelines and real-world feasibility [44]. Success in long-term metabolic management is contingent upon enhancing nutritional literacy, allowing for conscious dietary choices that align with the patient’s cultural and socioeconomic context rather than relying on rigid, prescriptive diets [14]. Key strategies include the simplification of dietary goals—utilizing familiar household measures and visual guides—to reduce cognitive load and foster early self-efficacy. Furthermore, the integration of behavioral self-regulation techniques, such as frequent self-monitoring, portion control, and the identification of environmental food triggers, is essential. Finally, the evidence suggests that sustained professional support and frequent educational reinforcement are the most robust predictors of long-term adherence, mitigating the common challenges associated with lifestyle modification in primary care settings [44].

These findings are consistent with other evidence underscoring the essential role of nutrition education in T2DM management. Mutagwanya et al. [11] reported that nutrition education interventions significantly improved dietary practices and lifestyle behaviors among individuals with T2DM, reinforcing that the educational process is a key determinant of clinical success.

Web-based platforms have proven to be a feasible and effective medium for delivering behavioral interventions in T2DM management, significantly enhancing nutritional knowledge and glycemic control. The integration of interactive components—such as personalized feedback, self-tracking, and peer support—promotes participant retention, thereby mitigating barriers to long-term engagement in chronic disease self-management [45].

The present study highlights that this simple approach, based on an internet-delivered nutritional education program within the context of type 2 diabetes, yielded multiple benefits over a short period of time, including reductions in medication use, glycated hemoglobin levels, body weight, and fasting plasma glucose. In contrast, the conventional approach currently disseminated to patients within the public healthcare system failed to demonstrate comparable positive outcomes.

### Study limitations

This study presents certain limitations related to the absence of information regarding the duration of T2DM diagnosis and the lack of waist circumference data in the *CG* medical records, which prevented the inclusion of this variable in comparative analyses.

A potential limitation of this study is the baseline imbalance observed between groups for body weight and HbA1c levels. Although the differences did not reach statistical significance (*p* > 0.05), the *IG* presented a higher mean body weight (approximately 10 kg more than the CG) and slightly lower baseline HbA1c levels. We acknowledge that the IG’s higher initial weight may have provided a greater margin for reduction, which could have influenced the magnitude of the observed improvements in glycemic control. Conversely, the slight weight gain in the *CG* (+ 2.5%) did not lead to significant changes in HbA1c. These baseline trends should be considered when interpreting the intergroup comparisons. Despite these baseline differences, the clinical improvements in the IG remained robust, suggesting that the intervention’s effect size transcends initial physiological variances.

Another limitation of this study was the lack of quantitative dietary intake assessment such as macronutrient distribution and total energy intake. Direct quantification of adherence to the prescribed dietary strategy was not feasible. However, the intervention was reinforced by a robust support system, including structured nutrition education, weekly messages, and continuous remote access to a nutritionist. While direct dietary quantification was not performed, the study utilized robust outcome assessment through HbA1c levels This biochemical marker minimizes reporting bias and maximizes the validity of the study findings. This pragmatic design reflects real-world primary care conditions, where success is primarily measured by clinical outcomes. Finally, the 16-week follow-up is relatively short; therefore, longer-term studies are required to confirm the potential for sustained remission associated with this educational and dietary approach. Future research should integrate objective dietary assessment methods and adherence evaluation to further validate these findings.

## Conclusion

The online educational intervention conducted by a nutritionist and based on a low-carbohydrate dietary approach proved to be more effective for managing T2DM in primary health care than conventional care during the same period, leading to superior short-term improvements in glycemic control, body weight, and the use of oral antidiabetic medications.

Despite the aforementioned limitations, these findings reinforce that clinical success is directly linked to the active role of nutritionists and the educational process, both enhanced by the use of digital technologies. The integration of structured nutrition education, a low-carbohydrate dietary strategy, and remote follow-up represents a scalable and promising approach for T2DM management, capable of improving adherence, expanding access, and contributing to the reduction of diabetes-related complications and public health care costs.

## References

[1] Brazil. Ministry of Health. National Diabetes Day: about 30 million consultations were performed in 2023. Brasília: Ministry of Health. 2024. Available from: https://www.gov.br/saude/pt-br/noticias/dia-nacional-do-diabetes-cerca-de-30-milhoes-de-atendimentos-foram-realizados-em-2023 Accessed 2024 Nov 9. in Portuguese.

[2] Sociedade Brasileira de Diabetes (SBD). Guidelines of the Brazilian Diabetes Society 2024–2025. São Paulo: Clannad. 2025. Available from: https://www.diabetes.org.br/profissionais/diretrizes-sbd Accessed 2024 Oct 25. [Article in Portuguese].

[3] Velayutham S, Panneerselvam S, Ramanathan K. Global diabetes epidemic: comprehensive insights into prevalence, risk factors, and emerging trends. J Diabetes Metab Disord. 2025;24(1):15. 10.1007/s40200-024-01357-y.39712337

[4] Neves RG, Tomasi E, Duro SMS, Saes-Silva E, Saes MO. Diabetes mellitus complications in Brazil: a national-based study, 2019. Cien Saude Colet. 2023;28(11):3183–90. 10.1590/1413-812320232811.07142023. in Portuguese. 37971002 10.1590/1413-812320232811.11882022

[5] Muzy J, Campos MR, Emmerick I, Silva RS, Schramm JMA. Prevalence of diabetes mellitus and its complications and characterization of gaps in health care based on research triangulation. Cad Saude Publica. 2021;37(5):e00076120. 10.1590/0102-311X00076120. in Portuguese. 34076095 10.1590/0102-311X00076120

[6] Skyler JS, Bakris GL, Bonifacio E, Darsow T, Eckel RH, Groop L, et al. Differentiation of diabetes by pathophysiology, natural history, and prognosis. Diabetes. 2017;66(2):241–55. 10.2337/db16-080. 27980006 10.2337/db16-0806 PMC5384660

[7] Hill MA, Zhang L, Sun Z, Jia G, Parrish AR, Sowers JR. Insulin resistance, cardiovascular stiffening and cardiovascular disease. Metabolism. 2021;119:154766. 10.1016/j.metabol.2021.154766. 33766485 10.1016/j.metabol.2021.154766

[8] Datta D, Kundu R, Basu R, Chakrabarti P. Pathophysiological hallmarks in type 2 diabetes heterogeneity (review). Diabetol Int. 2024;19(2):201–22. 10.1007/s13340-024-00783-w. 10.1007/s13340-024-00783-w PMC11954762 40166449

[9] Palasson RR, Paz EPA, Marinho GL, Pinto LFS, Teston EF, Gomes MA, et al. Quality of health care in primary care: perspective of people with diabetes mellitus. Rev Bras Enferm. 2023;76(5):e20220267. 10.1590/0034-7167-2022-0267. [Article in Portuguese]. 10.1590/0034-7167-2023-0008 PMC10561413 37820130

[10] Simão CCAL, Costa MB, Colugnati FAB, Paula EA, Vanelli CP, Paula RB. Quality of care of patients with diabetes in primary health services in Southeast Brazil. J Environ Public Health. 2017;2017:1709803. 10.1155/2017/1709803. [Article in Portuguese]. 10.1155/2017/1709807 PMC5654281 29129980

[11] Mutagwanya R, Nyago CM, Nakwagala FN. Effect of diabetes nutrition education on the dietary feeding practices and lifestyle of type 2 diabetic patients. Eur J Clin Nutr. 2022;76(2):270–6. 10.1038/s41430-021-00940-3. 34168295 10.1038/s41430-021-00940-3

[12] Athinarayanan SJ, Adams RN, Hallberg SJ, McKenzie AL, Bhanpuri NH, Campbell WW, et al. Long-term effects of a novel continuous remote care intervention including nutritional ketosis for the management of type 2 diabetes: a 2-year non-randomized clinical trial. Front Endocrinol (Lausanne). 2019;10:348. 10.3389/fendo.2019.00348. 31231311 10.3389/fendo.2019.00348 PMC6561315

[13] Goldenberg JZ, Day A, Brinkworth GD, Sato J, Yamada S, Jönsson T, et al. Efficacy and safety of low and very low carbohydrate diets for type 2 diabetes remission: systematic review and meta-analysis of published and unpublished randomized trial data. BMJ. 2021;372:m4743. 10.1136/bmj.m4743. 33441384 10.1136/bmj.m4743 PMC7804828

[14] Gortzi O, Dimopoulou M, Androutsos O, Vraka A, Gousia H, Bargiota A. Effectiveness of a nutrition education program for patients with type 2 diabetes mellitus. Appl Sci. 2024;14(5):2114. 10.3390/app14052114.

[15] Dening J, Mohebbi M, Abbott G, George ES, Ball K, Islam SMS. A web-based low carbohydrate diet intervention significantly improves glycaemic control in adults with type 2 diabetes: results of the T2Diet Study randomised controlled trial. Nutr Diabetes. 2023;13:12. 10.1038/s41387-023-00230-z. 37633959 10.1038/s41387-023-00240-8 PMC10460437

[16] Zhong B. How to calculate sample size in randomized controlled trial? J Thorac disease. 2009;1(1):51–4. 10.3978/j.issn.2072-1439.2009.12.01.011. 22263004 PMC3256489

[17] Yamada Y, Uchida J, Izumi H, Tsukamoto Y, Inoue G, Watanabe Y, et al. A non-calorie-restricted low-carbohydrate diet is effective as an alternative therapy for patients with type 2 diabetes. Intern Med. 2014;53(1):13–9. 10.2169/internalmedicine.53.1123. 24390522 10.2169/internalmedicine.53.0861

[18] Sato J, Kanazawa A, Makita S, Hatae C, Komiya K, Shimizu T, et al. A randomized controlled trial of 130 g/day low-carbohydrate diet in type 2 diabetes with poor glycemic control. Clin Nutr. 2017;36(4):992–1000. 10.1016/j.clnu.2016.07.003. 27472929 10.1016/j.clnu.2016.07.003

[19] Kawalkar U, Vidyasagar S, Fernandez K, Dixit JV, Deshpande M, Khadse A, et al. Evaluating diabetes-related nutrition knowledge and dietary beliefs among type 2 diabetes patients in Vidarbha region, Maharashtra: a mixed-method approach for insightful analysis. Front Nutr. 2025;12:1420662. 10.3389/fnut.2025.1420662. 40066153 10.3389/fnut.2025.1420662 PMC11891037

[20] Hite AH, Cavan D, Cywes R, Ede G, Fettke G, Lenzkes B et al. Clinical guidelines for therapeutic carbohydrate restriction. LowCarb USA; 2019. Available from: https://www.lowcarbusa.org/clinical-guidelines Accessed 2024 Oct 5.

[21] Aldafas R, Alharthi AS, Alharthi SS, Alqahtani SA, Alshahrani A, Alshahrani F, et al. The legacy effect of early HbA1c control on microvascular complications and hospital admissions in type 2 diabetes: findings from a large UK study. Ther Adv Endocrinol Metab. 2025;16:20420188251350897. 10.1177/20420188251350897. 40547904 10.1177/20420188251350897 PMC12181706

[22] Riddle MC, Cefalu WT, Evans PH, Gerstein HC, Nauck MA, Oh WK, et al. Consensus report: definition and interpretation of remission in type 2 diabetes. Diabetes Care. 2021;44(10):2438–45. 10.2337/dci21-0034. 34462270 10.2337/dci21-0034 PMC8929179

[23] Nakhleh A, Halfin E, Shehadeh N. Remission of type 2 diabetes mellitus. World J Diabetes. 2024;15(7):1384–9. 10.4239/wjd.v15.i7.1384. 39099816 10.4239/wjd.v15.i7.1384 PMC11292336

[24] Goday A, Bellido D, Sajoux I, Crujeiras AB, Burguera B, García-Luna PP, et al. Short-term safety, tolerability and efficacy of a very low-calorie-ketogenic diet interventional weight loss program versus hypocaloric diet in patients with type 2 diabetes mellitus. Nutr Diabetes. 2016;6(9):e230. 10.1038/nutd.2016.36. 27643725 10.1038/nutd.2016.36 PMC5048014

[25] Lean MEJ, Leslie WS, Barnes AC, Brosnahan N, Thom G, McCombie L, et al. Primary care-led weight management for remission of type 2 diabetes (DiRECT): an open-label, cluster-randomized trial. Lancet. 2018;391(10120):541–51. 10.1016/S0140-6736(17)33102-1. 29221645 10.1016/S0140-6736(17)33102-1

[26] Daly ME, Paisey R, Millward BA, Eccles C, Williams K, et al. Short-term effects of severe dietary carbohydrate-restriction advice in type 2 diabetes: a randomized controlled trial. Diabet Med. 2006;23(1):15–20. 10.1111/j.1464-5491.2005.01760.x. 16409560 10.1111/j.1464-5491.2005.01760.x

[27] Tay J, Luscombe-Marsh ND, Thompson CH, Noakes M, Buckley JD, Wittert GA, et al. Comparison of low- and high-carbohydrate diets for type 2 diabetes management: a randomized trial. Am J Clin Nutr. 2015;102(4):780–90. 10.3945/ajcn.115.112581. 26224300 10.3945/ajcn.115.112581

[28] Batista MES, Oliveira GF, Figueiredo ILA, Souza AC, Casimiro MRA, Oliveira LB. Cultural influence on adherence to diabetes treatment. Rev Ibero-Am Humanid Cienc Educ. 2025;11(10):4400–12. 10.51891/rease.v11i10.21722. [Article in Portuguese].

[29] Turnbull FM, Abraira C, Anderson RJ, Byington RP, Chalmers JP, Duckworth WC, et al. Intensive glucose control and macrovascular outcomes in type 2 diabetes. Diabetologia. 2009;52(11):2288–98. 10.1007/s00125-009-1470-0. 19655124 10.1007/s00125-009-1470-0

[30] Yan Y, Asemani S, Jamilian P, Changgen Y. The efficacy of low-carbohydrate diets on glycemic control in type 2 diabetes: a comprehensive overview of meta-analyses of controlled clinical trials. Diabetol Metab Syndr. 2025;17(1):341. 10.1186/s13098-025-01890-7. 40826376 10.1186/s13098-025-01890-7 PMC12362953

[31] American Diabetes Association. 2. Diagnosis and classification of diabetes: standards of care in diabetes—2025. Diabetes Care. 2025;48(Suppl 1):S27–S49. 10.2337/dc25-S002 PMC11635041 39651986

[32] Rafiullah M, Musambil M, David SK. Effect of a very low-carbohydrate ketogenic diet vs recommended diets in patients with type 2 diabetes: a meta-analysis. Nutr Rev. 2022;80(3):488–502. 10.1093/nutrit/nuab093. 34338787 10.1093/nutrit/nuab040

[33] Saslow LR, Mason AE, Kim S, Goldman V, Ploutz-Snyder R, Bayandorian H, et al. An online intervention comparing a very low-carbohydrate ketogenic diet and lifestyle recommendations versus a plate method diet in overweight individuals with type 2 diabetes: a randomized controlled trial. J Med Internet Res. 2017;19(2):e36. 10.2196/jmir.5806. 28193599 10.2196/jmir.5806 PMC5329646

[34] Ait-Ali N. Can digital technology revolutionize continuous education in diabetes care? J Diabetes Sci Technol 2024 Dec 3:19322968241298000. 10.1177/19322968241298000 10.1177/19322968241298000 PMC11571552 39535135

[35] Han Y, Cheng B, Guo Y, Wang Q, Yang N, Lin P. A low-carbohydrate diet realizes medication withdrawal: a possible opportunity for effective glycemic control. Front Endocrinol (Lausanne). 2021;12:779636. 10.3389/fendo.2021.779636. 34970224 10.3389/fendo.2021.779636 PMC8713744

[36] Caballero Mateos I, Ruiz Moral R, Torres Jiménez R, Quesada Varela VJ, Roldán Villalobos A, Palomo Llinares R, et al. Efficacy of a digital educational intervention for patients with type 2 diabetes mellitus: multicenter, randomized, prospective, 6-month follow-up study. J Med Internet Res. 2025;27:e60758. 10.2196/60758. 40209213 10.2196/60758 PMC12022518

[37] Dyson P. Low carbohydrate diets and type 2 diabetes: what is the latest evidence? Diabetes Ther. 2015;6(4):411–24. 10.1007/s13300-015-0136-9. 26446553 10.1007/s13300-015-0136-9 PMC4674467

[38] Assunção MCF, Santos IS, Valle NCJ. Controle glicêmico em pacientes diabéticos atendidos em centros de atenção primária à saúde. Rev Saúde Pública. 2005;39(2):183–90. 15895136 10.1590/s0034-89102005000200007

[39] Simão CCAL, Costa MB, Colugnati FAB, de Paula EA, Vanelli CP, de Paula RB. Quality of care of patients with diabetes in primary health services in Southeast Brazil. J Environ Public Health. 2017;2017:1709807. 10.1155/2017/1709807. 29129980 10.1155/2017/1709807 PMC5654281

[40] de Moraes HAB, Mengue SS, Molina M del, CB, Cade NV. Fatores associados ao controle glicêmico em amostra de indivíduos com diabetes mellitus do Estudo Longitudinal de Saúde do Adulto, Brasil, 2008 a 2010. Epidemiol Serv Saúde. 2020;29(3):e2018500. 10.5123/S1679-49742020000300017. 32555973 10.5123/S1679-49742020000300017

[41] Hallberg SJ, McKenzie AL, Williams PT, Bhanpuri NH, Peters AL, Campbell WW, et al. Effectiveness and safety of a novel care model for the management of type 2 diabetes at 1 year: an open-label, non-randomized, controlled study. Diabetes Ther. 2018;9:583–612. 10.1007/s13300-018-0373-9. 29417495 10.1007/s13300-018-0373-9 PMC6104272

[42] Alqassab O, Shuaibi S, Quqandi E, Dallak A, Almutairi A, Alharthi S, et al. Evaluating the impact of telemedicine on diabetes management in rural communities: a systematic review. Cureus. 2024;16(7):e64928. 10.7759/cureus.64928. 39035595 10.7759/cureus.64928 PMC11260063

[43] Costa LF, Sampaio TL, Moura L, Rosa RS, Iser BPM. Temporal trend and costs of hospitalizations with a primary diagnosis of diabetes mellitus in the Brazilian Unified Health System, 2011–2019. Epidemiol Serv Saude do Brasil. 2023;32(4):e20230006. 10.1590/S1679-49742023000400006.

[44] Newson L, Parody FH. Investigating the experiences of low-carbohydrate diets for people living with type 2 diabetes: a thematic analysis. *PLoS One*. 2022;17(8):e0273422. 10.1371/journal.pone.0273422. 10.1371/journal.pone.0273422 PMC9394793 35994442

[45] Ramadas A, Chan CKY, Oldenburg B, et al. Randomised-controlled trial of a web-based dietary intervention for patients with type 2 diabetes: changes in health cognitions and glycemic control. BMC Public Health. 2018;18:716. 10.1186/s12889-018-5640-1. 29884161 10.1186/s12889-018-5640-1 PMC5994015

